# The minimization of mechanical work in vibrated granular matter

**DOI:** 10.1038/srep28726

**Published:** 2016-07-04

**Authors:** James P. D. Clewett, Jack Wade, R. M. Bowley, Stephan Herminghaus, Michael R. Swift, Marco G. Mazza

**Affiliations:** 1 Max Planck Institute for Dynamics and Self-Organization, Am Faßberg 17, 37077 Göttingen, Germany; 2 School of Physics and Astronomy, University of Nottingham, Nottingham, NG7 2RD, United Kingdom

## Abstract

Experiments and computer simulations are carried out to investigate phase separation in a granular gas under vibration. The densities of the dilute and the dense phase are found to follow a lever rule and obey an equation of state. Here we show that the Maxwell equal-areas construction predicts the coexisting pressure and binodal densities remarkably well, even though the system is far from thermal equilibrium. This construction can be linked to the minimization of mechanical work associated with density fluctuations without invoking any concept related to equilibrium-like free energies.

Many-particle systems driven far from equilibrium, which occur abundantly in nature, technology, as well as in laboratory settings, often exhibit remarkable collective behaviour^1^,^2^,^3^,^4^,^5^,^6^,^7^,^8^, such as clustering, swarming, or laning. In spite of the importance of such phenomena, the search for underlying principles governing their dynamics and emerging patterns is still continuing^9^,^10^,^11^,^12^. Inspired by analogous problems in equilibrium thermodynamics, it has proven useful to study non-equilibrium steady states (NESS) which are characterized by time-independent, non-trivial macroscopic quantities (and their fluctuations), such as the pressure and densities in a phase separated system.

A paradigmatic system exhibiting such a NESS is a driven granular gas^13^,^14^,^15^,^16^. In its simplest form, a granular gas is a cloud of noncohesive, dissipative spherical particles, maintained in a steady state by a continuous external drive^17^,^18^. The degree of dissipation is quantified by the restitution coefficient, 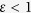, which denotes the ratio of the relative normal speeds of particles after/before a collision. Whenever *ε* < 1, one observes clustering in a freely cooling system, or phase separation if energy is continuously supplied. Recent work has demonstrated that loosely confined grains driven by a periodic external force can separate into liquid- and gas-like phases via spinodal decomposition^19^. A related two-dimensional system driven by a thermal wall also exhibits behaviour similar to the phase separation in a van der Waals gas^19^,^20^,^21^,^22^,^23^. Consequently, concepts borrowed from equilibrium statistical physics were used to describe its phase separation^20^,^21^,^22^,^24^. To date, investigations were limited to a parameter space very close to the elastic limit. In this limit it has been suggested that thermodynamic concepts are generally applicable, including a phenomenological ‘free energy’ based on a Landau expansion^21^. However, away from the elastic limit its behaviour is expected to differ, since some basic assumptions of equilibrium statistical physics, such as detailed balance, are no longer valid.

Here we investigate both experimentally and by computer simulations the phase separation behaviour of driven granular gases far away from the elastic limit, down to *ε* = 0.65. We demonstrate that not only can a Maxwell equal-areas construction predict the coexistence pressure and binodal densities remarkably well, but that such a construction can be applied, with reasonable accuracy, away from the critical point and for high dissipation. We argue that this construction can be traced to the minimisation of mechanical work associated with density fluctuations. Although the deviations from an exact Maxwell construction are small, we show their significance and provide a tentative interpretation.

## Results

### Pressure characteristics

**Figure 1:**
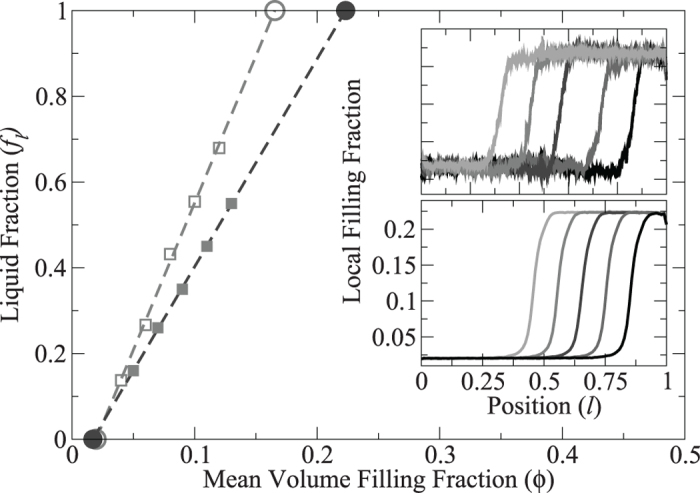
Main panel: The open and filled symbols represent the interface position as obtained from the experiment and the simulations, respectively. Dashed lines are linear fits to the data. Zero liquid fraction corresponds to gas density, *ϕ*_*g*_, unity liquid fraction represents liquid density, *ϕ*_*l*_ (circles). Insets: The top inset shows the mean grey level from photographs of the experiment, presented in arbitrary units. Data are shown for densities in the range 
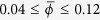
, increasing from right to left. The driving amplitude is *A* = 2.1*d*. The bottom inset shows the density profiles obtained from simulations. Data are shown for densities in the range 
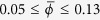
.

We study a system of approximately monodisperse spheres with diameter *d* = 610 *μ*m, confined between two horizontal plates separated by a distance of 10 mm, and driven vertically by a sinusoidal motion with amplitude *A*. We measured density profiles for the coexisting liquid-gas phase separation by using the long-cell apparatus described in the methods section. The results are shown in the insets in Fig. 1 and the corresponding liquid fractions are shown in the main panel. As the number of particles in the system (and hence the mean density 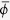) is increased, the volume of the liquid phase increases, moving the interface to the left. The densities *ϕ*_*l*_ and *ϕ*_*g*_ appear to be independent of 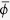. In the main panel the linear fits demonstrate that the system obeys a lever rule, 
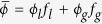
, where *f*_*l*_ is the liquid fraction and *f*_*g*_ is the gas fraction. The lever rule confirms that there is an intrinsic mechanism which selects the liquid and gas densities as intensive quantities of the system.

In order to get access to quantities which are not readily available experimentally, we performed time-driven molecular dynamics simulations of our system. We relax the system for a sufficient amount of time (ten seconds of simulated time was found appropriate) to ensure we have reached the steady state. The pressure, for example, is then determined by averaging both spatially and over ten distinct initial configurations, for ten seconds each. We define the pressure in the homogeneous regions to be the average of the trace of the horizontal components of the pressure tensor^25^. To obtain the distribution of local pressures, we coarse-grain the system using bins of length 5*d*. Varying the bin size in a sensible range does not affect the results reported here.

By simulating small sample cells with horizontal dimensions less than the liquid-gas interface width, phase separation can be suppressed. In this way the pressure can be calculated as a function of homogeneous quantities even under conditions for which a large system would phase separate. Periodic boundary conditions are used in the horizontal directions. This method has previously been employed to obtain the equation of state for granular gases^20^,^23^,^26^,^27^,^28^. However, recent work for systems in thermal equilibrium questions whether the non-monotonic pressure-volume curves obtained represent the equation of state for the system, or merely reflect finite size effects^29^,^30^. In the following paragraphs we will demonstrate that *P*(*v*) does indeed serve as the equation of state for our granular system.

**Figure 2:**
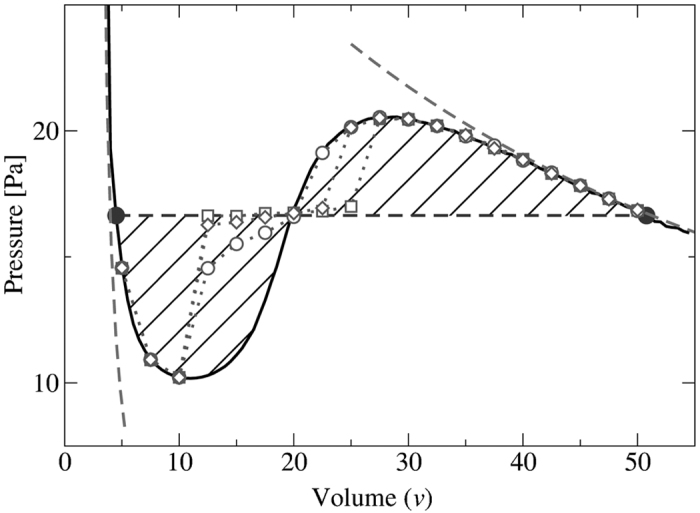
The solid line shows the pressure, *P*(*v*), calculated from simulations of a small square-base cell with side length *L* = 20*d*. In the unstable region, the pressure tends towards the dashed tie line which connects the binodal densities calculated in the large cell (*L* = 460*d*). The open circles, squares and diamonds show the pressure calculated in cells of length *L* = 80*d*, 120*d*, 160*d*, respectively, demonstrating the convergence to the large-cell limit. The grey, dashed asymptotes schematically indicate the low temperature branch (left) and high temperature branch (right).

Figure 2 shows the dependence of *P* on the dimensionless volume per particle, 
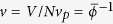
, in a small square-base cell of side *L* = 20*d* for *A* = 2.1*d* (solid line). As expected, the pressure exhibits a non-monotonic dependence on the volume, similar to that which is observed in a molecular fluid. Significantly, we find that for sufficiently *small* cells (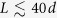) the calculated pressure is not a function of the system size.

For this system the pressure curve *P*(*v*) is not an isotherm. The physical origin of its non-monotonic shape is completely different to that of a molecular fluid. The granular gas has no attraction between the particles; instead, the dilute phase is ‘heated’ more effectively due to its intimate coupling to the vibrating walls, while the dense phase is strongly cooled by its frequent dissipative inter-particle collisions^23^,^31^. As a result, the non-monotonic behaviour in our system can be regarded as a crossover from a low “temperature” branch at high densities (left dashed curve in Fig. 2) to a high “temperature” branch at low densities (right dashed curve in Fig. 2). The open symbols indicate the pressure calculated in cells of different size, showing the convergence to the large-cell limit. We see that the region between the two extrema of *P*(*v*) is unstable against phase separation, resulting in a pressure corresponding to two-phase coexistence, P* (horizontal dashed line).

**Figure 3:**
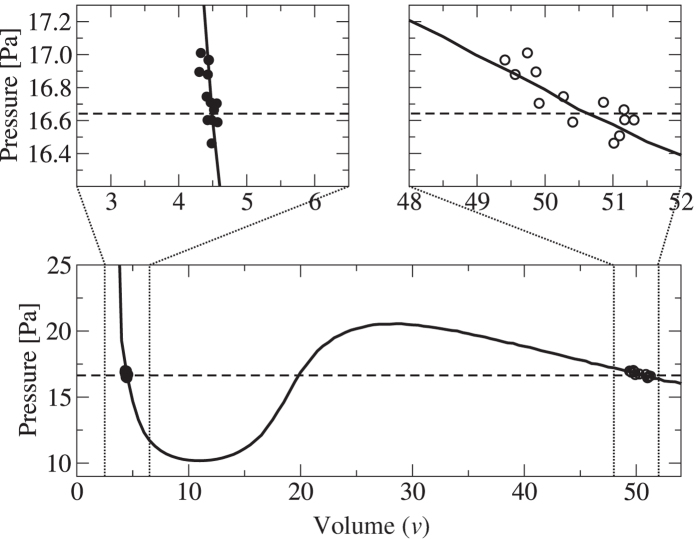
The pressure-volume curve calculated using a simulation of the small cell and an expanded view close to P*. The filled and open circles mark the averaged pressure and binodal volumes per particle for the liquid and gas phases respectively, obtained at different times. At each time, the average pressures in the two phases are equal to a good approximation.

Figure 3 shows *P*(*v*) as obtained from the small cell, juxtaposed with the pressure and volume per particle calculated using a long cell in which the system phase separates. Each circle represents an average over three driving cycles. Spatially the calculated pressure is approximately constant throughout the system, with a spatial mean value 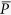. However, momentary imbalances in the energy injection and dissipation give rise to global pressure fluctuations around the temporal mean saturation pressure P* 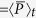. The corresponding densities fluctuate so as to remain on the pressure curve *P*(*v*), confirming that *P*(*v*) does indeed serve locally as the equation of state in both the liquid and gas phases.

**Figure 4:**
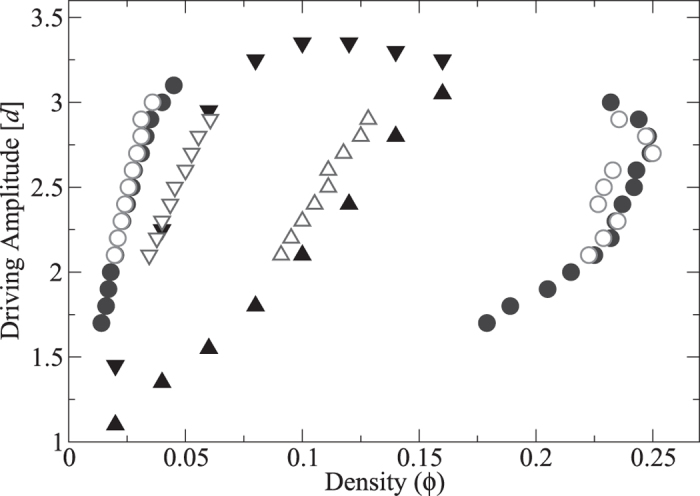
Phase diagram for the liquid-gas-like phase separation. The filled and open circles show the binodal points determined by the long cell simulations, and those predicted by *P*(*v*) and the equal-areas construction, respectively. The triangles show the spinodal points determined from the onset of phase separation in the long cell simulations and those predicted by the unstable region of *P*(*v*), respectively.

It is interesting to note that the horizontal dashed line in Fig. 2, corresponding to P*, creates two approximately equal areas bounded above and below by the curve *P*(*v*) (hatched). Figure 4 shows the spinodal and binodal lines determined directly from phase separation in the large cell (filled symbols), and the predictions made by using the small cell (open symbols). The open circles indicate the binodal points obtained from *P*(*v*) by assuming a Maxwell equal-areas construction holds at the equal-areas pressure, *P*^*e*^. The agreement is remarkable: in equilibrium thermodynamics the Maxwell construction is based on the minimization of the Gibbs free energy, and as such is not expected to hold here. This finding was reproduced to a similar degree of accuracy for all levels of dissipation investigated, down to *ε* = 0.65.

### Equal-areas construction

**Figure 5:**
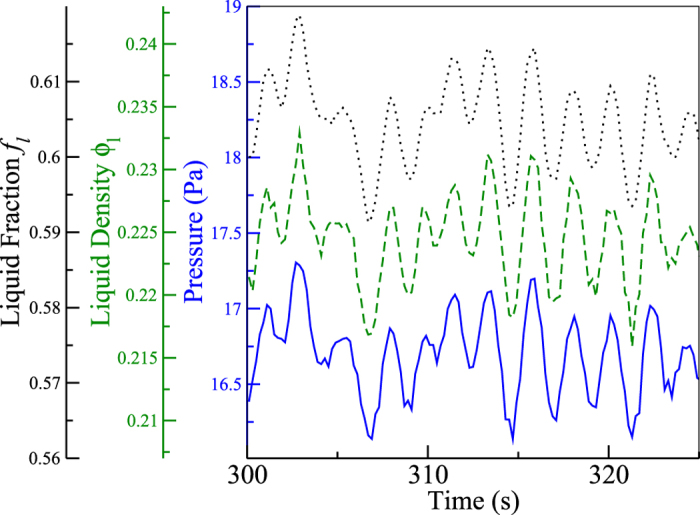
The solid line (blue) shows the instantaneous pressure 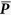, smoothed by a Gaussian filter over three driving cycles. The dashed line (green) shows the density of the liquid phase, and the dotted line (black) shows the liquid fraction, derived from the position of the interface.

In search of a physical basis for the equal-areas rule, we discuss the fluctuations we have observed in the phase-separated system, depicted in Fig. 5. The fluctuations in the liquid fraction, *f*_*l*_ (dotted), density of the liquid phase (dashed), and the mean pressure (solid) are strongly correlated because the volume and particle number are conserved and the system obeys an equation of state. Similar behaviour is observed in our experiments, which exhibit periodic fluctuations in the position of the interface.

When the pressure increases, the volume per particle decreases in each phase (as illustrated by Fig. 3) and both phases try to shrink. Since the total particle number and the volume of the cell are fixed, some particles in the liquid-like phase must be converted into the gas-like phase. There is a separation of time-scales between the frequency of pressure fluctuations (which are slow) and the frequency of collisions between particles (which are fast). We assume that the conversion of particles from one phase to the other occurs through a series of quasi-static states. As a consequence the mechanical work involved in such a change can be evaluated from a knowledge of *P*(*v*). At a pressure higher than *P*^*e*^, the conversion of particles from liquid-like to gas-like requires mechanical work to be done on these particles. The converse is also true: if the pressure drops, the specific volumes grow and particles must be converted from the dilute to the dense phase. At a pressure lower than *P*^*e*^ this too requires mechanical work. We refer to the additional energy to exchange particles between phases at a pressure different from *P*^*e*^ as *the residual mechanical work*. Fluctuations of the pressure away from *P*^*e*^ in either direction require residual mechanical work. In contrast to the quasi-static pressure variation, the granular temperature has fast dynamics; its value is governed by the evolution of the slow variables alone^21^.

**Figure 6:**
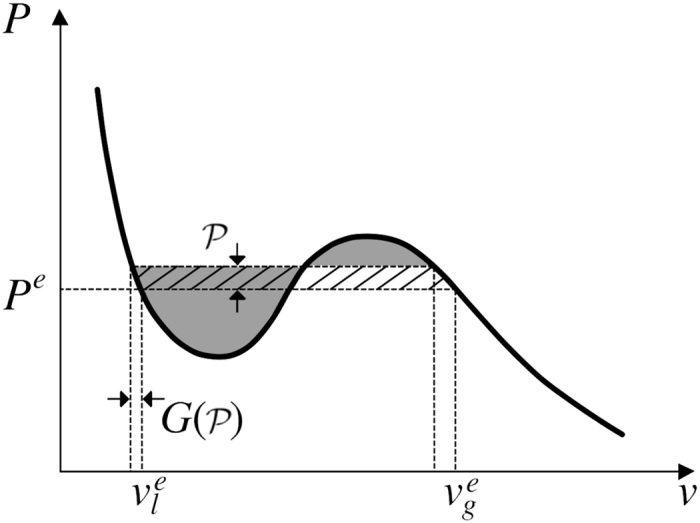
The difference between the left and right shaded areas shows the average mechanical work required to convert a particle from the dense phase to the dilute phase at a mean pressure 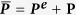. As *p*^*e*^ is the equal-areas pressure, this difference is equal to the total hatched area.

Taken all together these observations suggest the following purely mechanical model which uniquely identifies the saturation pressure and the binodal densities. Let the total volumes of the phases be *V*_*i*_ = *N*_*i*_*v*_*i*_, where *N*_*i*_ and *v*_*i*_ are the number of particles and the specific volumes, respectively, for *i* ∈ {*l, g*}. For any fluctuation, the total number and volume of the particles is fixed, and *v*_*l*_ and *v*_*g*_ change so that 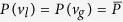, the instantaneous pressure. For our granular gas we have shown that the pressure oscillates, and define 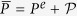. The corresponding change in the specific volume is defined to be 
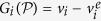
, where 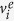 are the specific volumes at the equal-areas pressure. It is straightforward to determine 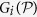 directly from the equation of state. The difference between the left and right shaded areas in Fig. 6 illustrates the average amount of work required to convert one particle from the dense to the dilute phase at a mean pressure 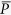. Only if *P*^*e*^ is the equal-areas pressure is this difference equal to the hatched area and given by


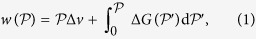


where 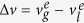 and 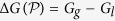.

For the system to obey the equation of state, *P*(*v*), 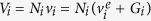. In any fluctuation 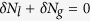 and 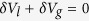, so that


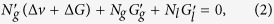


where the primes indicate derivatives with respect to 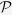, and the 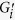
s are minus the compressibilities in each of the two phases. By defining 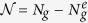 we can rewrite this as


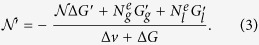


Therefore the total residual mechanical work done for a finite fluctuation, 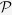, is given by


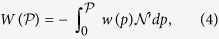


which to leading order reduces to


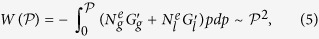


as 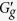 and 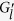 are both negative. Since 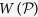 is quadratic in 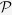 any fluctuation that shifts the pressure away from *P*^*e*^ while keeping the volumes per particle on *P*(*v*) requires residual mechanical work to be done. We hypothesise that, because of dissipation, the system tries to minimise the residual mechanical work, and 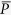 fluctuates around *P*^*e*^ as observed in simulations.

It is interesting to quote from Maxwell’s discussion of the equal-areas rule in equilibrium systems: “*Since the temperature has been constant throughout, no heat has been transformed into work*”^32^. In our system the temperature is not constant throughout an expansion, yet, because the system tries to remain on the equation of state, an equal-areas rule still appears to be applicable to a good approximation. It is based solely on the minimization of the residual mechanical work.

**Figure 7:**
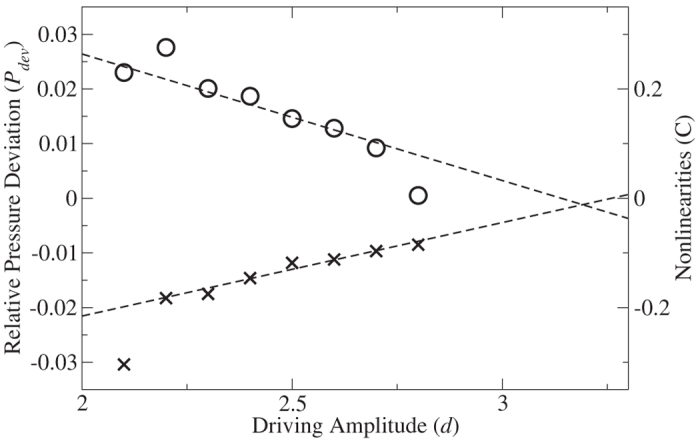
Deviation. The circles show the relative difference between the pressure obtained from an equal-areas construction and the pressure at coexistence calculated in the phase-separated system; the dashed-line is a linear fit to guide the eye. The crosses show 
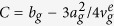
.

The equal-area construction described above is able to predict the coexisting pressure remarkably well, typically to within less than 2%. However, it is not exact. In Fig. 7 we show the pressure deviation *P*_*dev*_ = (*P*^*e*^ − P*)/*P*^*e*^ as a function of amplitude. As the amplitude increases, the deviation decreases and approaches zero at the critical point, *A* = 3.2 *d*, as would be expected. We attribute the deviation from the equal-areas construction to the shape of 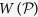 and to the fluctuations observed in experiment and simulation. If 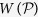 were a symmetric function, the mean pressure P* would be expected to be equal to the minimum of *W*, namely *P*^*e*^. However, in general 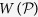 is not symmetric and the mean pressure is not equal the pressure at the minimum.

To quantify the asymmetry, we model the curvature of *P*(*v*) close to the Maxwell points as 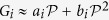. By substituting for *G*_*i*_ in Eq. 1 and Eq. 3 and expanding Eq. 4 as a power series in 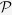, we find that the 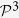 term vanishes when


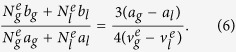


Details of the calculation are given in the supplemental material. Since 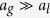, 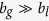 and 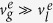, the nonlinearity should vanish when 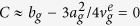. The crosses in Fig. 7 show *C* calculated from small-cell simulations for different driving amplitudes. Both *P*_*dev*_ and *C* extrapolate to zero at the critical point and increase in magnitude as the driving amplitude decreases, indicating that deviations from the Maxwell construction are caused by an increase in the nonlinearity.

## Discussion

So far we might conclude that the minimization of residual mechanical work, as outlined above, holds generally, which would suggest that an analogue of a free energy functional could be obtained for our system, e.g., by integration of *P*(*v*). This would be in line with conclusions drawn previously from results obtained much closer to the elastic limit^21^. However, this is not the complete picture.

One has to appreciate that the Maxwell construction is only an approximation, based on the assumption that the slow, mechanical variables (the pressure and the density), are completely decoupled from the fast kinetic variables (the temperature, dissipation and energy injection from the walls). As such any free energy analogue derived from *P*(*v*) will be predictive only for the mechanical variables. Conversely, the minimisation principle that we have obtained will not be useful to describe nor predict any aspects of the kinetic variables. Since it is the kinetic variables which give rise to the non-monotonic pressure characteristic^23^, the free-energy analogue cannot describe the NESS in its entirety.

Finally, we note that a number of studies on shear flow of granules have observed a non-monotonic dependence of the pressure on the volume. Campbell^33^ and later Alam & Luding^34^ showed that the stress tensor dependence on the solid fraction has a characteristic **U**-shape with asymptotes at both the low density limit and at the density of the shearable limit (random-close packing). We remark that the physical origin of the non-monotonicity is, however, different. In simulations of shear flow^33^,^34^ the stresses diverge at low density because the few collisions taking place at low density must dissipate increasingly large amounts of energy; at high density the stresses grow because they reach the limit where considerable stresses are necessary to initiate or maintain the shear flow^35^. The different natures of the contributions to the stress tensor at low and high filling fractions are reflected in the separation of the stress tensor in streaming and collisional contributions, which bring about the low and the high filling fraction divergences, respectively. Conversely, the steady state in our results arises from a balance of different rates of dissipation in the liquid-like and gas-like phases. The crossover from the low temperature, dense phase to the high temperature, dilute phase in the presence of an interface between them engenders the non-monotonic behaviour of *P*(*v*) in Fig. 2. Furthermore, the kinetic theories of Jenkins & Savage^36^ and Lun *et al*.^37^, e.g., are based on the assumption that fluctuations (that is, gradients in the density, temperature, and velocity) are small. This assumption is strongly violated in our conditions (see Fig. 5). The determination of the equation of state for strongly driven systems is then called for.

It would nevertheless be interesting to compare our results with the framework provided by the kinetic theory of granular systems^36^,^38^,^39^,^40^,^41^,^42^,^43^,^44^. The interplay of the dynamical fluctuations with the boundary driving, which generates the results presented here, should prove an important testing bed for generalizations of the kinetic theory. This is left for future work.

## Methods

### Experiments

Our experimental apparatus is very similar to that used previously^23^. Glass particles were confined between parallel horizontal plates in a long, thin cell. The particles were sieved and selected under a microscope to obtain a sample of approximately monodisperse spheres with diameter *d* = 610 *μ*m. The cell was constructed from a lower plate of 3 mm thick, anodized aluminum and a top plate of 3 mm thick glass. The plates were separated by 10 mm high aluminum walls which also confined the particles horizontally such that the internal length, width and height of the cell were 280 mm, 10 mm, and 10 mm, respectively. The cell was driven sinusoidally in the vertical direction, with variable amplitude, *A*. The driving frequency was kept fixed at 60 Hz. The mean density is defined as 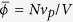, where *N* is the number of particles, *v*_*p*_ is the volume of a single particle and *V* is the internal volume of the cell. Care was taken to ensure that the cell was level prior to each experimental run.

### Computer simulations

In addition to our experiments, we have also carried out time-driven molecular dynamics simulations. The simulations have previously been shown to accurately capture the physics of the system under study^19^,^23^. The particles are modelled as monodisperse soft-spheres with diameter *d* = 610 *μ*m. Dissipation is included by a normal coefficient of restitution, *ε* (implemented using a linear-spring and dash-pot damping)^45^. The effects of tangential forces and rotational degrees of freedom are neglected because these have been shown to have minor impact on the physics of the system^46^,^47^,^48^. The simulated cell has dimensions 460*d* × 20*d* × 16.4*d*, thus closely resembling the experimental system. Reflecting boundary conditions on the short walls of the cell are used in order to study a single interface between two coexisting phases. On the long walls, periodic boundary conditions are employed. The results presented in this paper are for *ε* = 0.8, matching well with the experiment, but similar results are found for the rather wide range of 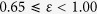, that is, all coefficients of restitution for which the liquid-gas phase separation can be uniquely identified.

## Additional Information

**How to cite this article**: Clewett, J. P. D. *et al*. The minimization of mechanical work in vibrated granular matter. *Sci. Rep.* **6**, 28726; doi: 10.1038/srep28726 (2016).

## Supplementary Material

Supplementary Information
