# Supplementary material for: The minimization of mechanical work in vibrated granular matter
Source: Sci Rep. 2016 Jul 4;6:28726. doi: 10.1038/srep28726 (PMC4931499; doi:10.1038/srep28726)
Supplement: Supplementary Information [file srep28726-s1.pdf]

Supplemental Material:
The minimization of mechanical work in vibrated granular matter

James P.D. Clewett,[1] Jack Wade,[2] R. M. Bowley,[2] Stephan Herminghaus,[1] Michael R. Swift,[2] and Marco G. Mazza[1]

[1]*Max Planck Institute for Dynamics and Self-Organization, Am Faßberg 17, 37077 Göttingen, Germany*
[2]*School of Physics and Astronomy, University of Nottingham, Nottingham, NG7 2RD, United Kingdom*

MECHANICAL WORK

In this section we repeat the derivation of the equations in the main text, for clarity here we include more explicit steps. Let the total volumes of the phases be $V_i = N_i v_i$, where N_i and v_i are the number of particles and the specific volumes respectively, for $i \in \{l, g\}$. If the system has an instantaneous mean pressure slightly away from the equal-areas pressure, P^e, given by $\bar{P} = P^e + \mathcal{P}$, then for the system to remain on the equation of state, $P(v)$, the specific volumes must vary by an amount $G(\mathcal{P}) = v_i - v_i^e$, where v_i^e are the volumes corresponding to P^e

Figure 1 shows the measured equation of state and the equal-areas pressure, P^e. A and D label the shaded areas, B and C label the hatched areas. The average work done to exchange one particle from the dense to the dilute phase is given by the difference between the shaded areas. From the construction illustrated in the figure, $A - B = C + D$ so that the difference between the shaded regions is given by $A - D = B + C$, that is the total hatched region. This can be written as

$$w(\mathcal{P}) = \Delta v \mathcal{P} + \int_0^{\mathcal{P}} \Delta G(P) \mathrm{d}P, \tag{S1}$$

where $\Delta v = v_g^e - v_l^e$ and $\Delta G(\mathcal{P}) = G_g - G_l$.

For the system to obey the equation of state: $V_i = N_i v_i = N_i(v_i^e + G_i)$, therefore

$$V_i' = N_i'(v_i^e + G_i) + N_i G_i', \tag{S2}$$

where the symbol of prime indicates the derivative with respect to $\mathcal{P}$, and G_i' are the compressibilities of the two phases. For any finite fluctuation $\delta N_l + \delta N_g = 0$ and $\delta V_l + \delta V_g = 0$, so that Eq. S2 becomes

$$N_g'(\Delta v + \Delta G) + N_g G_g' + N_l G_l' = 0. \tag{S3}$$

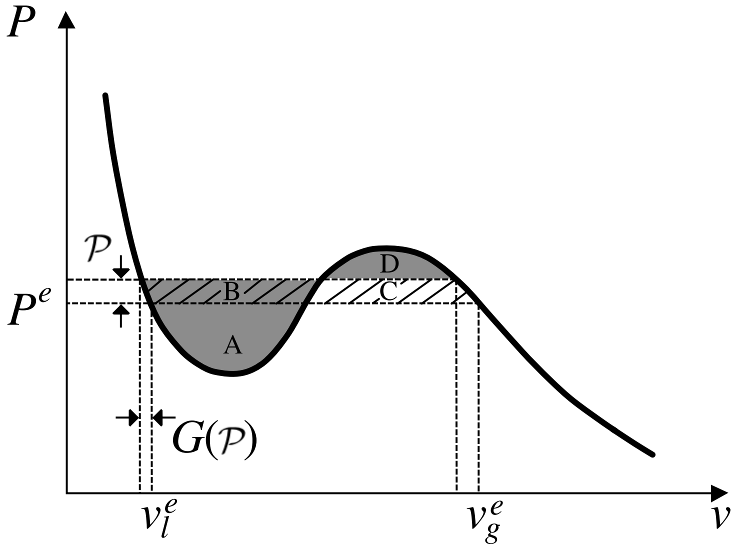

FIG. 1. A sketch of the equation of state, $P(v)$, for a granular gas under external energy feed. The quantities marked by the superscript e correspond to the steady-state pressures and volumes. The labels A, B, C, D mark useful areas in our construction.

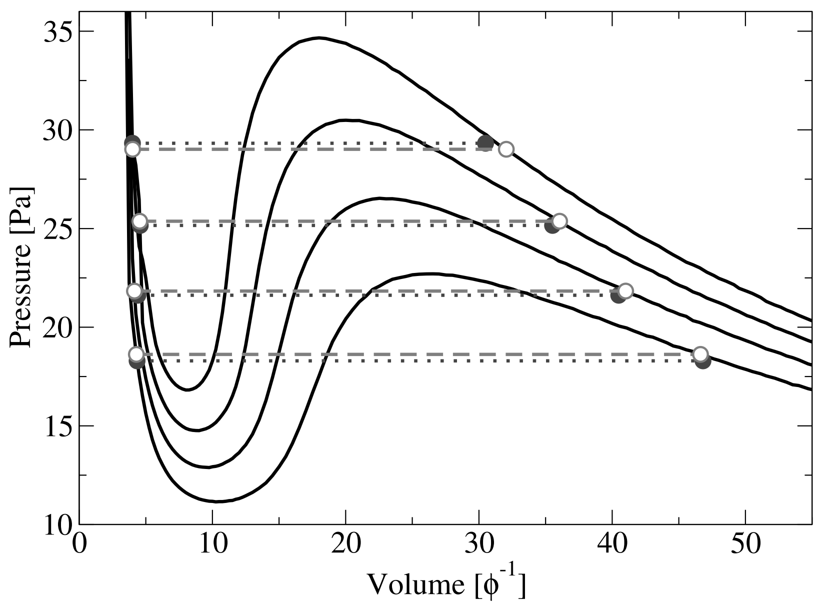

FIG. 2. The solid lines show the pressures calculated in small cells for driving amplitudes in the range $2.2d \leq A \leq 2.8d$. The filled circles show the corresponding binodal densities and the pressures calculated in long, thin systems. The open circles show the pressure and densities predicted using an equal-areas construction.

By defining $\mathcal{N} = N_g - N_g^e$ (or equivalently $-\mathcal{N} = N_l - N_l^e$), we can rewrite this as

$$\mathcal{N}' = -\frac{\mathcal{N}\Delta G' + N_g^e G_g' + N_l^e G_l'}{\Delta v + \Delta G}. \tag{S4}$$

The total work done for a finite fluctuation, $\mathcal{P}$, is given by

$$W(\bar{P}) = \int_0^{\mathcal{P}} w(P)\mathcal{N}' dP. \tag{S5}$$

From Fig. 2 it is clear that the time averaged saturation pressure calculated in long cell deviates slightly from the equal-areas rule, that is $P^* \neq P^e$. In order to understand the origin of this deviation, it is necessary to examine the contribution of the higher order terms in the integrand of Eq. S5. To do this we model the curvature of $P(v)$ close to the Maxwell points as $G_i \approx a_i\mathcal{P} + b_i\mathcal{P}^2$. By substituting for G_i in Eq. S1 we obtain

$$w(\mathcal{P}) = \Delta v\mathcal{P} + \frac{\Delta a}{2}\mathcal{P}^2 + \frac{\Delta b}{3}\mathcal{P}^3, \tag{S6}$$

where $\Delta a = a_g - a_l$ and $\Delta b = b_g - b_l$. Substituting for G_i in Eq. S4 we find

$$\mathcal{N}' = -\frac{\mathcal{N}(\Delta a + 2\mathcal{P}\Delta b) + \left[N_g^e a_g + N_l^e a_l + 2\mathcal{P}(N_g^e b_g + N_l^e b_l)\right]}{\Delta v + \Delta a\mathcal{P} + \Delta b\mathcal{P}^2}. \tag{S7}$$

In order to obtain an overview of the different powers of $\mathcal{P}$ we expand the denominator, $D(\mathcal{P})$, appearing in Eq. S7 so that

$$D(\mathcal{P}) \approx \frac{1}{\Delta v}\left[1 - \frac{\Delta a}{\Delta v}\mathcal{P} + \left[\left(\frac{\Delta a}{\Delta v}\right)^2 - \frac{\Delta b}{\Delta v}\right]\mathcal{P}^2\right]. \tag{S8}$$

If we also expand $\mathcal{N} \approx \alpha\mathcal{P} + \beta\mathcal{P}^2$, then we write Eq. S7 in the form

$$\alpha + 2\beta\mathcal{P} = -D(\mathcal{P})\left[(\alpha\mathcal{P} + \beta\mathcal{P}^2)(\Delta a + 2\mathcal{P}\Delta b) + A + 2B\mathcal{P}\right], \tag{S9}$$

where $A = N_g^e a_g + N_l^e a_l$ and $B = N_g^e b_g + N_l^e b_l$.

Comparing coefficients of $\mathcal{P}$ in Eq. S9 we find that

$$\mathcal{N} = -\frac{1}{\Delta v}\left(A\mathcal{P} + \left(B - \frac{\Delta a}{\Delta v}A\right)\mathcal{P}^2\right). \tag{S10}$$

As such we can write down the integrand of Eq. S5 in the form

$$w(\mathcal{P})\mathcal{N}' = -A\mathcal{P} - \left(2B - \frac{3}{2}\frac{\Delta a}{\Delta v}A\right)\mathcal{P}^2. \tag{S11}$$

It is now clear that the first nonlinear term is determined by the expression

$$2B - \frac{3}{2}\frac{\Delta a}{\Delta v}A = 2(N_g^e b_g + N_l^e b_l) - \frac{3(a_g - a_l)}{2(v_g^e - v_l^e)}(N_g^e a_g + N_l^e a_l), \tag{S12}$$

which vanishes when

$$\frac{N_g^e b_g + N_l^e b_l}{N_g^e a_g + N_l^e a_l} = \frac{3(a_g - a_l)}{4(v_g^e - v_l^e)}, \tag{S13}$$

Eq. 6 in the main text.

By including higher order terms we have shown that the equal-area rule is only an approximation, the different compressibilities $-\partial v/\partial P\ |_{v=v_{l,g}^e}$ give rise to an asymmetry in the potential $W(\mathcal{P})$, such that on the average, the saturation pressure is slightly offset from the equal-areas pressure. The calculated systematic variation of the inequality provides additional evidence that the minimization of the residual mechanical work defines the binodal densities.

PRESSURE CALCULATIONS

This section contains each of the various pressure measurements discussed in the main text. A number of different calculations are used to obtain the pressure, therefore this supplementary material is provided for the avoidance of confusion. In this report, all calculations are taken from the horizontal plane, parallel to the driving plates.

The pressure tensor $\mathbf{P}$ is calculated using the virial equation:

$$P_{\alpha\beta} = \frac{1}{2}m\sum_{i=1}^{N_p} v_{i\alpha}v_{i\beta} + \sum_{i=1}^{N_p}\sum_{j=i+1}^{N_p} f_{i\alpha}\delta_{j\beta}, \tag{S14}$$

where i and j are the particle labels, α and β are dimensional labels in the x and y directions, m is the mass of a particle, and $v_{i\alpha}$ is the instantaneous velocity of the particle i in the α direction. The quantity $f_{i\alpha}$ is the instantaneous force on particle i in the α direction due to an overlap $\delta_{j\beta}$ with a neighbouring particle, j, in direction β. It is only non-zero when particle i overlaps with particle j. The scalar pressure is the trace of the horizontal components of the pressure tensor, $P(v) = \frac{1}{2}(P_{xx} + P_{yy})$.

The scalar pressure is, to a very good approximation, always homogeneous in space. As such, the spatially averaged pressure is used to represent the instantaneous pressure in the system. This is given by the average of the two-dimensional scalar pressure, $\bar{P} = \langle P(v)\rangle$. The spatially averaged pressure $\bar{P}$ oscillates approximately periodically in time. Therefore the mean saturation pressure is taken to be the time average of the spatial average, $P^* = \langle \bar{P}\rangle_t$.

The equal-areas pressure P^e is the pressure which defines a tie-line enclosing equal areas of the equation of state calculated in a small cell, $P(v)$. The difference between the ideal equal-areas pressure, and the instantaneous spatial average is therefore given by $\mathcal{P} = P^e - \bar{P}$.
